# A simple method for semi-random DNA amplicon fragmentation using the methylation-dependent restriction enzyme MspJI

**DOI:** 10.1186/s12896-015-0139-7

**Published:** 2015-04-11

**Authors:** Hiroshi Shinozuka, Noel O I Cogan, Maiko Shinozuka, Alexis Marshall, Pippa Kay, Yi-Han Lin, German C Spangenberg, John W Forster

**Affiliations:** Department of Economic Development, Jobs, Transport and Resources, Biosciences Research Division, AgriBio, Centre for AgriBioscience, 5 Ring Road, La Trobe University Research and Development Park, Bundoora, Victoria 3083 Australia; Dairy Futures Cooperative Research Centre, Bundoora, Australia; School of Applied Systems Biology, La Trobe University, Bundoora, Victoria 3086 Australia

**Keywords:** Second-generation sequencing technology, Genotyping-by-sequencing, 5-methylcytosine, Restriction endonuclease, DNA shearing

## Abstract

**Background:**

Fragmentation at random nucleotide locations is an essential process for preparation of DNA libraries to be used on massively parallel short-read DNA sequencing platforms. Although instruments for physical shearing, such as the Covaris S2 focused-ultrasonicator system, and products for enzymatic shearing, such as the Nextera technology and NEBNext dsDNA Fragmentase kit, are commercially available, a simple and inexpensive method is desirable for high-throughput sequencing library preparation. MspJI is a recently characterised restriction enzyme which recognises the sequence motif CNNR (where R = G or A) when the first base is modified to 5-methylcytosine or 5-hydroxymethylcytosine.

**Results:**

A semi-random enzymatic DNA amplicon fragmentation method was developed based on the unique cleavage properties of MspJI. In this method, random incorporation of 5-methyl-2’-deoxycytidine-5’-triphosphate is achieved through DNA amplification with DNA polymerase, followed by DNA digestion with MspJI. Due to the recognition sequence of the enzyme, DNA amplicons are fragmented in a relatively sequence-independent manner. The size range of the resulting fragments was capable of control through optimisation of 5-methyl-2’-deoxycytidine-5’-triphosphate concentration in the reaction mixture. A library suitable for sequencing using the Illumina MiSeq platform was prepared and processed using the proposed method. Alignment of generated short reads to a reference sequence demonstrated a relatively high level of random fragmentation.

**Conclusions:**

The proposed method may be performed with standard laboratory equipment. Although the uniformity of coverage was slightly inferior to the Covaris physical shearing procedure, due to efficiencies of cost and labour, the method may be more suitable than existing approaches for implementation in large-scale sequencing activities, such as bacterial artificial chromosome (BAC)-based genome sequence assembly, pan-genomic studies and locus-targeted genotyping-by-sequencing.

**Electronic supplementary material:**

The online version of this article (doi:10.1186/s12896-015-0139-7) contains supplementary material, which is available to authorized users.

## Background

Massively parallel short-read sequencing technologies have become commonly used not only for *de novo* genome sequencing, but also for a wide range of biological purposes, such as resequencing and large-scale genotyping studies. Fragmentation at random nucleotide locations is an essential component of library construction for the various short-read sequencing instruments [1], through delivery of multiple read initiation points in template molecules. Sequence information may then be decoded through computational assembly of the short reads. Physical shearing is recommended by the manufacturers of all second-generation massively parallel DNA sequencing systems, due to the high reproducibility and randomness of fragmentation. However, the process is likely to require the use of dedicated instruments. The Nextera technology (Illumina, California, USA) and the NEBNext dsDNA Fragmentase kit (New England Biolabs, Massachusetts, USA) are alternative random DNA fragmentation methods which require only standard laboratory instruments [2-4]. The Nextera technology uses a transposon-transposase combination for random fragmentation of template DNA and attachment of transposon ends at the cleaved sites, permitting subsequent PCR amplification and sequencing. With the NEBNext dsDNA Fragmentase kit, double-stranded template DNA is fragmented in two sequential steps: nicks are enzymatically introduced into DNA, which is then cleaved at the nicked sites. These enzyme-based methods, however, require DNA sample preparation (buffer replacement and DNA concentration adjustment) for effective digestion, and the size of products is sensitive to both DNA sample quality and reaction duration, all of which require optimisation for each sample in order to achieve the desired outcome.

MspJI is a recently characterised modification-dependent endonuclease [5]. The enzyme was isolated from *Mycobacterium* sp. JLS and recognises sites containing the sequence motif CNNR (R = G or A nucleotides) when the first base is a 5-methylcytosine (5^m^C) or 5-hydroxymethylcytosine, and cleaves DNA at N_12_/N_16_ bases distant from the modified cytosine on the 3′-side. Enzyme activity is enhanced by short double-stranded DNA that includes the MspJI recognition site (and so acts as an enzyme activator). Digestion of genomic DNA with the MspJI enzyme generates fragments 32-34 bp in length, containing ^m^CpG or ^m^CNG sites central to the fragment. Methylation status of the human genome has been analysed through sequencing of such fragments [6]. Due to these unique features as a methylation-dependent restriction enzyme with adjacent non-specific cleavage activity, MspJI is expected to be highly useful for DNA modification and epigenomic studies [7].

FspEI and LpnPI are also recently characterised modification-dependent endonucleases, derived from *Frankia* sp. EAN1pec and *Legionella pneumophila* Philadelphia 1, respectively [5]. The sequence recognition sites for FspEI and LpnPI are 5’-CC-3’ and 5’-CCDG-3’ (D = A, G or T nucleotides), respectively, when the second base is a 5^m^C or 5-hydroxymethylcytosine. Similar to MspJI, activity of the two enzymes is stimulated by short DNA templates containing the recognition site, and the enzymes produce DNA fragments with 5’- termini including cohesive ends. These two enzymes hence provide potential alternatives to MspJI as a tool for DNA fragmentation.

The present study describes a simple and inexpensive method for generation of semi-randomly fragmented DNA from amplicon templates. DNA amplicons with randomly-incorporated 5-methyl-2’-deoxycytidine-5’-triphosphate (5-methyl-dCTP) were synthesised with DNA polymerase, and then digested with the MspJI restriction enzyme. The size range of the MspJI-digested fragments was capable of control through optimisation of 5-methyl-dCTP concentration. A purification procedure is unnecessary for DNA digestion with MspJI, which permits high-throughput sequencing library preparation. Short DNA fragments were also generated from a range of templates with a whole genome amplification kit based on activity of the Φ29 DNA polymerase, using the same methodology. Illumina sequencing libraries with inserts of 200 or 550 bp in length were successfully prepared using the MspJI-digested DNA, and were processed on the Illumina MiSeq platform.

## Results and discussion

### DNA amplification with 5-methyl-dCTP and MspJI digestion

**Figure 1:**
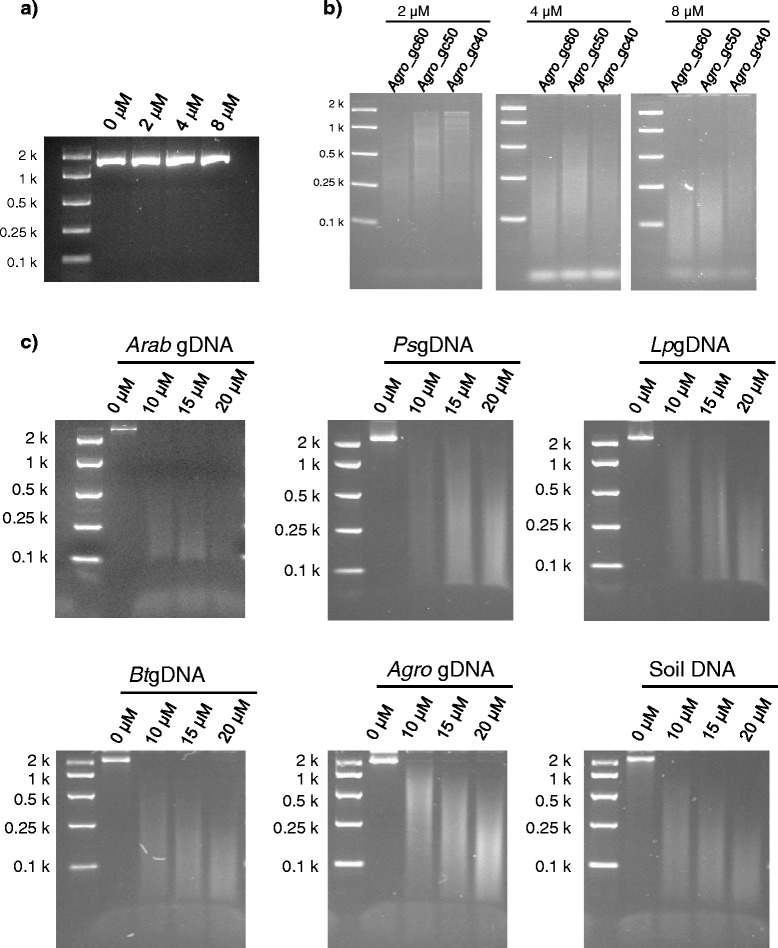
MspJI-enzymatic digestion of 5^m^C-containing PCR and Φ29 products. (**a**) DNA fragments amplified with the locus-specific PCR primers for the *Agro*_gc50 sequence under the presence of 5-methyl-dCTP (0, 2, 4 or 8 μM). (**b**) MspJI-digested DNA fragments derived from PCR products with each locus-specific primers and *Agro* gDNA as DNA template. Molar concentration denotes the 5-methyl-dCTP-concentration in PCR solution. (**c**) MspJI-enzymatic digestion of Φ29 enzyme-amplified DNA with randomly incorporated 5^m^C from a range of DNA templates. 0, 10, 15 and 20 μM denote final concentrations of 5-methyl-dCTP in the REPLI-g WGA mixture.

PCR amplification in the presence of 5-methyl-dCTP was performed with locus-specific primers and *Agrobacterium tumefaciens* genomic DNA (*Agro* gDNA) as template (Additional file 1). No significant difference in yield of PCR amplicon due to 5-methyl-dCTP concentration levels (final concentrations of 2, 4, or 8 μM in the PCR solution) was observed, based on the results of agarose gel electrophoresis, which was consistent with the previous studies (Figure 1a) [8,9]. The PCR amplicons of the *Agro*_gc40, *Agro*_gc50 and *Agro*_gc60 sequences, of which G/C content ratios were 40, 50 and 60%, respectively, were digested using the MspJI enzyme to identify the consequence of 5^m^C-containing DNA cleavage in the size-resolution range afforded by a 2.5% (w/v) agarose gel (Figure 1b). The size range of the digested DNA largely depended on concentration of 5-methyl-dCTP in the amplification solution, such that a lower range was obtained by a higher 5-methyl-dCTP concentration. Size range was also related to G/C content ratio, such that smaller DNA fragments were identified when amplicons with a higher ratio were digested (Figure 1b). This is probably due to the presence of at least one C-G base pair in the MspJI recognition site (5^m^CNNR), which will produce a small bias in incidence toward regions of higher G/C content ratio.

The *Agro*_gc50 sequence was also amplified with four types of DNA polymerase. The sequence was successfully amplified with all polymerases in the presence of 5-methyl-dCTP, and the amplicons were digested with MspJI. No significant differences in size range were observed, suggesting that a variety of DNA polymerases may be used for the proposed DNA fragmentation method (Additional file 2). A further characterisation of MspJI enzymatic activity indicated that components of the PCR solution do not significantly affect activity of the MspJI enzyme when diluted in the reaction mixture; the MspJI-mediated digestion of amplicons is completed within 4 hours; and the digestion result is independent of input DNA amount, when performed in an appropriate volume of reaction mixture (Additional file 3).

Whole genome amplification (WGA) was performed using the QIAGEN REPLI-g mini kit in the presence of 5-methyl-dCTP (10 to 20 μM) with genomic DNA samples from *Arabidopsis thaliana* (L.) Heynh. ecotype Columbia (*Arab* gDNA), a field pea (*Pisum sativum* L. subsp. *sativum* var. *arvense* (L.) Poir.) genotype (*Ps*gDNA), a perennial ryegrass (*Lolium perenne* L.) genotype (*Lp*gDNA), a bovine (*Bos taurus* L.) genotype (*Bt*gDNA), *Agro* gDNA, and a DNA sample from soil harvested in South Australia (Soil DNA) as templates. Amplified product was visualised on an agarose gel, revealing no significant differences in DNA amplification due to variation of 5-methyl-dCTP concentration. The amplified DNA was digested with MspJI (Figure 1c). Similar size distribution patterns were detected across varying 5-methyl-dCTP concentrations: a majority of DNA fragments from the 20 μM 5-methyl-dCTP-containing solutions was shorter than 250 bp, and a proportion of DNA fragments from 10 μM 5-methyl-dCTP-containing solutions was close to 1,000 bp, or larger, in size.

### Massively parallel sequencing of MspJI-digested templates

**Figure 2:**
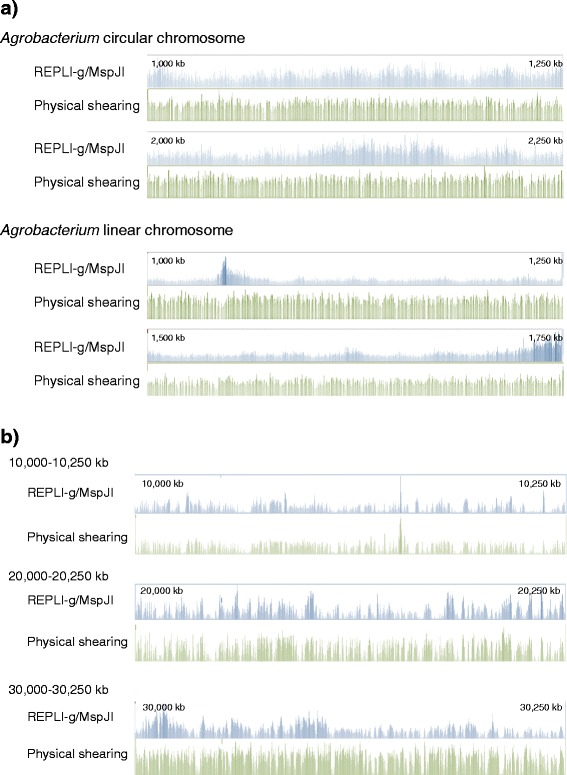
Illumina MiSeq short read-sequencing results of the libraries constructed from MspJI-digested and physically sheared DNA. The sorted alignment was visualised using the Tablet viewers. (**a**) Alignment results of Φ29 enzyme-amplified *Agro* gDNA-derived reads and physically sheared *Agro* gDNA-derived reads to the reference *Agrobacterium* genome sequences for the 1,000-1,250 and 2,000-2250 kb regions of the circular and linear chromosomes. (**b**) Alignment results of the *Arabidopsis* genome with the MspJI-enzymatic fragmentation and physical shearing methods. Read coverage depth for the 10,000-10,250, 20,000-20,250 and 30,000-30,250 kb regions of chromosome 1 was visualised.

For exemplification of the effects on distribution of fragments generated with the present method, sequencing libraries were prepared from *Agro* gDNA and *Arab* gDNA, using the MspJI-based and standard physical fragmentation methods (Additional file 4). The libraries were sequenced on the Illumina MiSeq platform. Totals of 1,380,029 and 1,219,389 reads derived from Φ29 enzyme amplification were aligned with the *Agrobacterium* circular and linear chromosomes (Figure 2a, Table 1). From the physically sheared DNA, totals of 546,292 and 496,662 reads were aligned with the circular and linear chromosomes, respectively. When the short reads from the MspJI-digested and physically sheared DNA were aligned, 99.99% and 99.8% of the reference *Agrobacterium* genome sequence was covered, respectively. When the sequencing reads derived from *Arab* gDNA were aligned with the reference sequence, 3-5 million reads were assigned to each chromosome (Figure 2b, Table 1). Average depths of coverage from the enzymatically fragmented and physically sheared libraries were 43.3 and 53.3 times, respectively, which covered 97.9% and 99.3% of the reference *Arabidopsis* genome sequence. The results of resequencing activities suggested that MspJI successfully generates DNA templates with a moderate level of random fragmentation.

**Table 1:** **Result of** ***Agrobacterium*** **and** ***Arabidopsis*** **genome resequencing using the two different DNA fragmentation methods**

**Species**	**Fragmentation method**	**Chromosome**	**Number of reads**	**Ratio of coverage (%)**	**Average coverage depth (time)**
*Agrobacterium*	REPLI-g/MspJI	Circular	1,380,029	99.9	64.3
		Linear	1,219,389	99.9	77.8
	Physical shearing	Circular	546,292	99.8	27.4
		Linear	496,662	99.9	34.1
*Arabidopsis*	REPLI-g/MspJI	1	4,611,463	97.3	36.4
		2	4,759,134	98	57.7
		3	4,702,657	98.3	47.9
		4	3,088,231	98	39.9
		5	4,222,266	98	37.8
	Physical shearing	1	5,116,095	98.9	41.5
		2	6,143,569	99.5	75
		3	6,083,236	99.5	62.4
		4	3,776,130	99.5	49.3
		5	4,980,370	99.4	45.1

Ratio of coverage denotes the value for specific nucleotides located on each chromosome.

**Figure 3:**
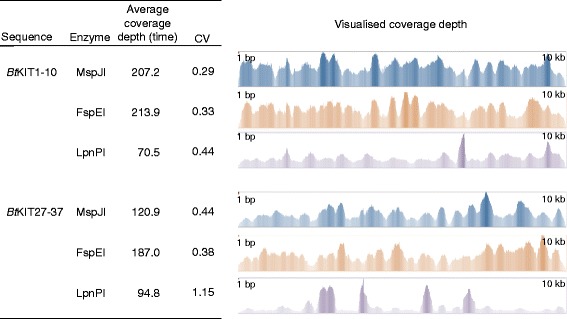
Short read-sequencing results of the libraries constructed from MspJI, FspEI and LpnPI-digested DNA. Read coverage depth was visualised using the Tablet viewer.

For a further exemplification of the effects on distribution of fragments generated with a methylation-dependent restriction enzyme, the *Bt*KIT1-10 and *Bt*KIT27-37 sequences were amplified from *Bt*gDNA, using the Roche Expand Long Range dNTPack kit in the presence of 7.5, 15 and 60 μM 5-methyl-dCTP (Additional file 1). Amplicons from the 7.5, 15 and 60 μM 5-methyl-dCTP reactions were digested with MspJI, FspEI or LpnPI (Additional file 5). Illumina sequencing libraries were prepared from the enzyme-digested DNA and sequenced on the MiSeq platform (Additional file 4). Totals of 3566-10,972 reads were aligned with the reference sequences, covering each nucleotide position of the reference. CVs for each data set were between 0.29 and 1.11 (Figure 3). The read alignment result indicated that the read distribution from the LpnPI-digested library was more skewed than those from the other libraries, and there was no large difference in the CVs between the MspJI- and FspEI-digested libraries. The average frequencies of potential MspJI-, FspEI- and LpnPI-recognition sites were once in every 4, 8 and 51.2 bp, respectively. This alignment result suggests that although the read distribution pattern depends on the frequency of the recognition sites, an incidence of once in every 8 bp is sufficient to generate reads with relatively even distribution.

### Potential application 1: sequencing of BAC clones

Although high-throughput DNA sequencing technologies have delivered a cost-efficient whole-genome shotgun sequencing method for those species with large genome sizes, information from BAC-based genomic libraries is valuable for effective DNA sequence assembly [10]. Sequence information from BAC-ends is commonly used for *de novo* assembly of large genomes [11,12]. The BAC-end sequencing procedure, however, requires a large investment, as it depends on the Sanger sequencing method [13]. A simple sequencing method for BAC clones using high-throughput sequencing technologies is described here.

**Figure 4:**
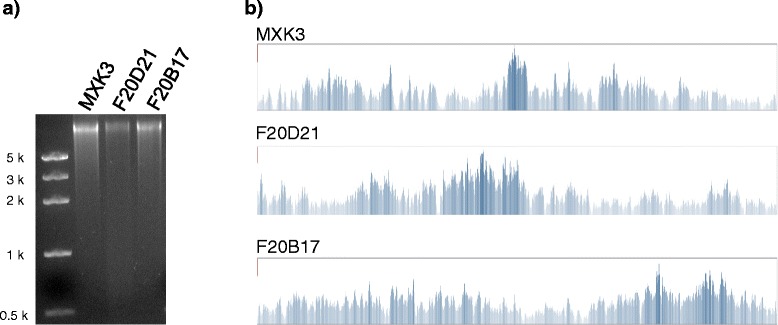
BAC clone sequencing based on the MspJI-based DNA fragmentation approach. (**a**) Amplified BAC DNA directly from glycerol stocks, using the Φ29 enzyme. In the amplification solution, 30 μM 5-methyl-dCTP were included. (**b**) Resequencing results of the BAC clones. Read coverage depth for each clone was visualised on the Tablet software.

BAC DNA was amplified in the reaction mixture of the REPLI-g mini kit, in which 30 μM 5-methyl-dCTP were included (Figure 4a, Additional file 6). The amplicons were digested with MspJI. A sequencing library was prepared from the MspJI-digested DNA and processed on the Illumina MiSeq platform to generate 395,498-558,276 reads for each sample. Totals of 27,585-86,861 reads (7.0-15.6%) were aligned with the reference, which covered over 99.6% of the reference sequences (Figure 4b, Table 2). The alignment result indicated that 81%-86% of reads were derived from the *Escherichia coli* (*E. coli*) genome. When a subset of 100,000 reads was aligned, around 99% of the reference BAC insert sequences were covered an average redundancy of 21-27 times. A single run of the Illumina MiSeq platform may generate up to 50 million reads, permitting individual sequencing of a whole 384-well plate of BAC clones with moderate depth of read coverage. With the Illumina MiSeq platform, a single clone may be analysed at the cost of US$7.2 (Additional file 7).

**Table 2:** **Resequencing results of the BAC clones**

		**All reads**				**100,000 read subset**		
**BAC name**	**BAC length**	**Total reads**	**Aligned reads (%)**	**Ratio of coverage (%)**	**Average coverage depth (time)**	**Aligned reads**	**Ratio of coverage (%)**	**Average coverage depth (time)**
MXK3	81 kb	395,498	27,585 (7.0%)	99.6%	82.3	7086	98.6%	21.3
F20D21	143 kb	558,276	86,861 (15.6%)	99.8%	149.4	15382	99.2%	26.5
F20B17	90 kb	478,300	46,409 (9.7%)	>99.9%	126.2	9173	99.9%	24.9

Ratio of coverage denotes the value for specific nucleotides located on each clone. A subset of 100,000 reads was obtained using the Seqtk software.

### Potential application 2: whole genome amplification and sequencing of bacterial and fungal genomes

High-throughput DNA sequencing technologies also provide an efficient method for pan-genome studies, especially for bacterial and fungal species [14,15]. Due to high levels of genomic diversity, a substantial number of bacteria or fungal strains must, however, be sequenced in order to define both core- and pan-genome constituents, and so a high-throughput library preparation method is required. Previously, direct WGA from fungal tissues was suggested as an efficient DNA sample preparation method [16]. A combination of the WGA and MspJI-based DNA fragmentation methods may permit high-throughput library processing.

**Figure 5:**
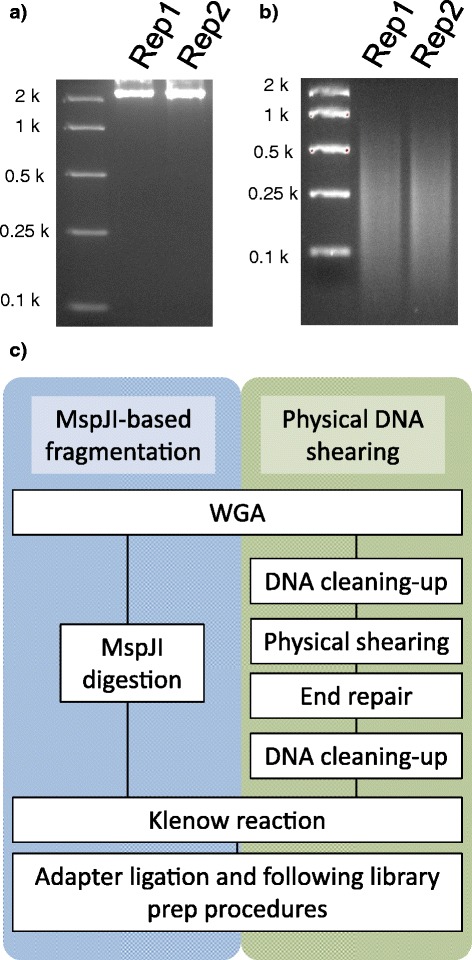
Sequencing of the fungal endophyte genome with the MspJI-based DNA fragmentation method. Two independent experiments (rep1 and rep2) were performed using the single endophyte strain. (**a**) WGA of the endophyte genome with the Φ29 enzyme in the presence of 5-methyl-dCTP (X μM). (**b**) MspJI digestion of the endophyte genome-derived amplicons. (**c**) Flowchart of the MspJI- and physical shearing-based library prep procedures from WGA products.

Genomic DNA from the perennial ryegrass-associated endophyte (*Epichloë festucae* var. *lolii* syn. *Neotyphodium lolii*) was amplified from a section of fungal mycelium (Figure 5a). In the multiple displacement amplification (MDA) reaction mixture, 15 μM 5-methyl-dCTP was included, and the amplified DNA was digested with MspJI (Figure 5b). A sequencing library was prepared from the MspJI-digested DNA and processed on the Illumina MiSeq platform. Totals of 2.7 million reads were generated, and 81,237 and 59,445 reads were aligned with the reference contig, which represent 1.6 and 1.3 Mb sections, respectively, of a genome 30 Mb in length (Table 3). A previous study reported that WGA was successfully achieved from as few as 24 fungal spores, which contribute to significantly reduced durations of DNA sample preparation [16]. DNA fragmentation with MspJI does not require several procedures subsequent to WGA, and so may enhance efficiency of the WGA-based sequencing library preparation (Figure 5c).

**Table 3:** **Resequencing results of the ryegrass endophyte genome**

**Reference**		**Alignment**				
**UI**	**Length (bp)**	**Aligned reads**	**Ratio of coverage (%)**	**Average coverage depth (time)**	**Max coverage depth (time)**	**Mismatch (%)**
gi|347366940	1,598,175	81,237	88.7	10.1	749	1.5
gi|347366939	1,323,136	59,445	86.7	8.8	304	1.4

UI denotes the unique identifier of the NCBI GenBank. Short read sequencing data from two experiments (rep1 and rep2) were combined for read alignment.

### Potential application 3: sequencing of PCR amplicons

Massively parallel sequencing technologies have permitted whole genome re-sequencing in a cost-effective manner [17]. Subsequently, genome-wide association studies (GWASs) have identified DNA polymorphisms that are correlated with trait-specific variation [18]. The numbers of relevant DNA polymorphisms identified through GWASs have, however, been relatively small [17,18]. Identification of trait locus variation-related DNA polymorphisms could hence be usefully followed by conversion into specific PCR-based markers, permitting locus-targeted genotyping over larger numbers of individuals [19,20].

**Figure 6:**
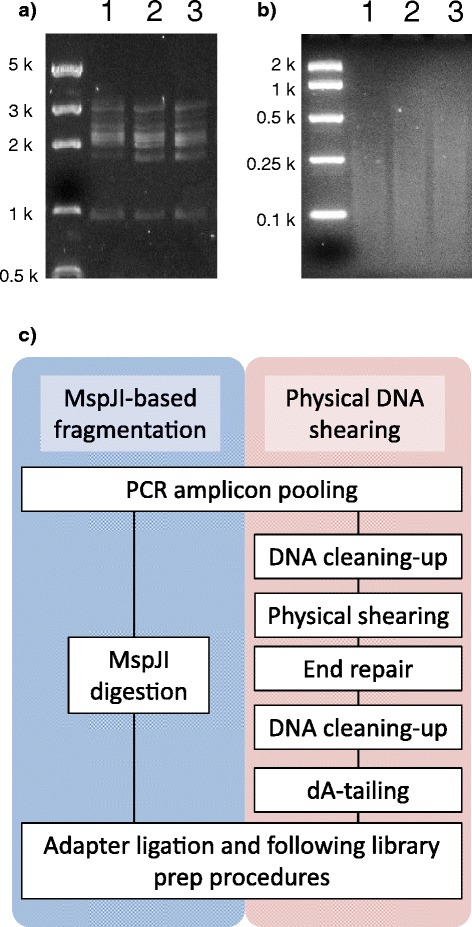
Resequencing of PCR amplicons with the MspJI-based DNA fragmentation method. (**a**) Pooled PCR amplicons containing 5^m^C. Locus-specific amplification was performed for each sequence independently. (**b**) MspJI digestion of the PCR amplicons. (**c**) Flowchart of the MspJI- and physical shearing-based library prep procedures from PCR amplicons.

Through PCR with 5-methyl-dCTP, candidate sequences for genes involved in the perennial ryegrass flowering signaling pathway were amplified from the DNA samples of the p150/112 F_1_ genetic mapping population (Figure 6a) [21]. The PCR amplicons were digested with MspJI, and sequencing library was prepared for the Illumina MiSeq platform (Figure 6b, Additional file 8). The sequencing reads were aligned against the references, and SNPs, which could be utilised for genetic linkage mapping studies, were identified in 7 of the sequences (Additional file 9). In the *Lp*CO and *Lp*FT sequence, no variation was identified between the two parental-derived sequence haplotypes. Due to the residual presence of heat-resistant DNA polymerase and dNTPs which could perform end-filling and adenine-tailing reactions during heat-inactivation of MspJI, the DNA fragments from restriction enzyme digestion could be directly used for DNA adapter ligation (Figure 6d). Cost assumption analysis suggested that the MspJI digestion method would be less expensive than the previously described cost-reduced physical shearing method (Additional file 10) [22]. Due to a lesser requirement for capital expenditure on equipment, the MspJI digestion method would become further cost-competitive when sample number is less than 100,000 [22,23]. The operator-specific time for the MspJI digestion method was expected to be 40% less than the physical shearing method (Additional file 11).

## Conclusions

The present study has reported a novel method for DNA fragmentation using the MspJI enzyme, which has been exemplified for a range of template types. A DNA sample preparation procedure, such as buffer replacement and DNA concentration adjustment, is not essential for MspJI digestion, which permits a simple DNA library preparation procedure from amplicons. A modified method involving combined use with other modification-dependent restriction enzymes may improve the random nature of the fragmentation. The size range of the resulting fragments was capable of control through adjustment of the 5-methyl-dCTP concentration in the amplification reaction solution, providing various fragment ranges from <100 bp to >2 kb. The method may hence be applicable for recombinant DNA purposes other than second-generation massively parallel short read sequencing technologies. Development of a computational methodology may improve sequencing efficiency with this method, through optimisation of 5-methyl-dCTP concentration and prediction of coverage for each nucleotide.

## Methods

### DNA sample preparation

DNA samples were prepared using the QIAGEN DNeasy kit (QIAGEN, Hilden, Germany) (*Arab* gDNA, *Ps*gDNA and *Lp*gDNA) and the Gentra PUREGENE® DNA Purification Kit (QIAGEN) (*Bt*gDNA), BioRad AquaPure Genomic DNA Kit (Bio-Rad Laboratories, CA, USA) (*Agro* gDNA). The Soil DNA sample was extracted with the MoBio Powersoil kit (MoBio, CA, USA), following a modified protocol [24]. DNA concentrations were adjusted to 5-30 ng/μl in the TE buffer using the NanoDrop system (Thermo Fisher Scientific, MA, USA).

#### DNA amplification with 5-methyl-dCTP

Locus-specific primers were prepared for amplification of the *Agro*_gc40, *Agro*_gc50 and *Agro*_gc60, *Bt*KIT1-10 and *Bt*KIT27-37 sequences (Additional file 1), and PCR was performed with Phusion Hot Start DNA polymerase (Thermo Fisher Scientific) and The Expand Long Range dNTPack (Roche Applied Science, Penzberg, Germany), following the manufacturer’s protocol. 5-methyl-dCTP (TriLink, CA, USA) was added to the PCR mixture at final concentrations of from 0 to 60 μM. WGA was performed using the REPLI-g mini kit (QIAGEN). Following the manufacturer’s protocol, 2.5 μl DNA (12.5-75 ng) was denatured with the D1 solution for three minutes, and then neutralised with the N1 solution. The amplification was performed in the reaction mixture with the presence of from 0 to 100 μM 5-methyl-dCTP (final concentrations) at 30°C for 16 hours. After incubation, the DNA polymerase was heat-inactivated, and the products were diluted with the same amount of water.

#### Restriction enzyme digestion

The amplified DNA (5 μl) was digested with 3 U of the MspJI, FspEI or LpnPI restriction enzyme (NEB) following manufacturer’s protocol. After incubation at 37°C for 4-16 hours, the enzyme was heat-inactivated at 70°C for 20 minutes.

#### Illumina sequencing library construction

Sequencing libraries were constructed with the TruSeq DNA Sample Preparation kit (Illumina) or NEBNext® DNA Library Prep Master Mix Set for Illumina® (NEB) with modifications. End-filling and adenine-tailing reactions for the MspJI-digested REPLI-g products were performed with the A-Tailing Mix or Klenow Fragment (3’ → 5’ exo^-^). For the PCR amplicons with the Expand Long Range dNTPack, the heat-inactivation procedure of the restriction enzyme also permitted end-filling of the restriction enzyme-digested fragments in the presence of the activated heat-resistant DNA polymerase and dNTPs. Blunt-ended DNA was purified with the AMPure XP bead kit (Life Technologies, CA, USA) and was used for the adenine-tailing reaction of the NEB kit. The ‘Purify Ligation Products’ process based on agarose gel electrophoresis (Illumina kit) was not performed. Following ligation of the DNA adapter index, the ligated DNA was purified with the AMPure XP bead kit and enriched through PCR. The size range of enriched DNA fragments was determined with the Agilent 2100 Bioanalyzer and Agilent DNA 1000 Kit (Agilent Technologies, CA, USA). The sequencing library was quantified with the KAPA Library Quantification Kit (Kapa Biosystems, MA, USA), following the manufacturer’s protocol.

Following the standard procedure, sequencing libraries from the *Agro* gDNA and *Arab* gDNA templates were prepared with the Illumina and NEB library prep kits, respectively. For these libraries, genomic DNA was fragmented with the S2 focused-ultrasonicator system (Covaris, MA, USA) following the manufacturer’s protocol.

#### Sequencing library preparation from BAC-containing clone glycerol stocks

*Arabidopsis* BAC clones (MIXK3, F20D21 and F20B17) were amplified with the QIAGEN REPLI-g mini kit. Glycerol stock (4 μl) of a BAC-containing *E. coli* clone was mixed with the Buffer D1 (4 μl) and incubated on ice for 5 minutes. The Buffer N1 (8 μl) was added into the sample and mix by stirring with a tip. The sample was incubated at room temperature for 3 minutes. The reaction mixture, consists of 5.8 μl REPLI-g Reaction Buffer, 0.2 μl REPLI-g Mini DNA Polymerase and 0.4 μl 5-methyl-dCTP (750 μM), was added into 3.4 μl denatured sample, and the reaction mixture was incubated at 30°C for 16 hours. The amplicons (5 μl) were digested with MspJI and the end-filling reaction was performed with Klenow Fragment (3’ → 5’ exo^-^). Sequencing adopter ligation was performed with T4 ligase (NEB) and ligated DNA was cleaned with AMPure XP bead solution (x0.8) to exclude short DNA. DNA fragments were subsequently enriched through PCR with the phusion DNA polymerase Kit. Small fragments (<500 bp), in which fraction *E. coli* genome-derived fragments were highly prevalent, were removed through size-selection with AMPure XP bead solution (x0.6). The sequencing library was characterised with the Agilent 2100 Bioanalyzer, Agilent DNA 1000 Kit, and the Qubit® Fluorometer (Life Technologies), following the manufacturer′s protocols.

#### Whole genome amplification and sequencing library preparation from perennial ryegrass-derived endophyte mycelium

Ryegrass endophyte genomic DNA was amplified with the QIAGEN REPLI-g mini kit. A section (2-3 mm^2^) of endophyte mycelium was placed into 6 μl PBS solution. The Buffer D2 (7 μl) was added into the sample and incubated on ice for 10 minutes, following mixing by a vortex. The Stop Solution was, then, added and mixed by a vortex. The reaction mixture, consisting of 29 μl REPLI-g Reaction Buffer, 1 μl REPLI-g Mini DNA Polymerase and 1 μl 5-methyl-dCTP (750 μM), was added into 9 μl of denatured sample, and the sample was incubated at 30°C for 16 hours. The amplicons (5 μl) were digested with MspJI and the sequencing library preparation was performed following the BAC clone sequencing protocol. The PCR-enriched DNA was cleaned with AMPure XP bead solution (x0.8). The sequencing library was characterised with the Agilent 2100 Bioanalyzer and Qubit® Fluorometer.

#### Sequencing library preparation from PCR amplicon

Locus-specific primers for the *Lp*AP1, *Lp*CO, *Lp*CRY1, *Lp*FLD, *Lp*FT, *Lp*LHY, *Lp*PHYC, *Lp*TOC1 and *Lp*Vrn5 sequences, and the MyFi™ DNA Polymerase kit (BIOLINE), which contains DNA polymerase that lacks 3′ → 5′ exonuclease activities, were used for PCR amplification (Additional file 1). The DNA samples of the p150/112 F_1_ mapping population were used as DNA templates. In the PCR solution, 8 μM 5-methyl-dCTP was included [25]. The PCR products were pooled for MspJI digestion (37°C for 4 hours) and MspJI was inactivated through incubation at 70°C for 20 mins. The heat-inactivation procedure also permitted end-filling and adenine-tailing of the MspJI-digested fragments in the presence of the activated heat-resistant DNA polymerase and dNTPs. Sequencing adapter ligation was performed with T4 ligase and ligated DNA was cleaned with AMPure XP bead solution (x0.8) to remove short DNA. DNA fragments were, then, enriched through PCR and the product was cleaned with AMPure XP bead solution (x0.8). The sequencing library was characterised with the 2200 TapeStation system (Agilent) and Qubit® Fluorometer.

#### Massively parallel sequencing and read assembly

The Illumina MiSeq sequencing platform was used to generate sequence output for sequencing libraries with the Illumina MiSeq Reagent Kit v2 or v3. Reads were attributed by the use of sample-specific DNA bar codes. The generated sequence reads were then checked for quality and integrity using a custom PERL script. Any reads with more than 3 consecutive Ns or more than 3 nucleotides with PHRED score ≤ 20 or a median PHRED score < 20 or a read length <50 nucleotides were trimmed or removed. The specific DNA sequence reads were then reference-aligned to the respective amplicon, *Agrobacterium* C58 (NCBI accession numbers: AE007869 and AE007870) [26] or *Arabidopsis* Columbia sequence [27] (http://www.arabidopsis.org/index.jsp). Reference alignments were performed using the BWA software package and then converted to a sorted BAM file using the SAMtools software package (http://samtools.sourceforge.net/). The Seqtk software package was used for generation of a subset sequence data (https://github.com/lh3/seqtk). Alignment of the sequencing reads to the reference sequences was visualised using the Tablet software [28].

### Availability of supporting datasets

The data sets supporting the results of this article are included within the article and its additional files.

## Additional files

Additional file 1:
**DNA sequence name, sequence of locus-specific PCR primers, size and G/C content ratio of PCR amplicons, and type of DNA polymerase used for PCR amplification.**
Additional file 2:
**MspJI-enzymatic digestion of 5** ^**m**^ **C-containing PCR amplicons.** The *Agro*_gc50 sequence was amplified with the Immolase™, MangoTaq™ *Pfu* and Phusion Hot Start polymerases and digested with MspJI. Molar concentration denotes the 5-methyl-dCTP-concentration in the PCR solution. Additional file 3:
**Characterisation of MspJI enzymatic activity.** Through PCR, 8 μM 5^m^C-containing amplicons were generated, and the products were cleaned with the AMPure kit. A range of DNA amounts (100, 200, 500, and 1,000 ng) was used for MspJI digestion, of which 50 ng digested DNA was visualised on an agarose gel. Additional file 4:
**Illumina MiSeq sequencing libraries constructed with the MspJI, FspEI or LpnPI-digested amplicons.** Sharp peaks with red and blue triangles show size standards (15 bp and 1,500 bp, respectively) of the Agilent DNA 1000 Kit. A peak between 200 and 1,500 bp represents the size distribution of DNA fragments of the constructed DNA library. Additional file 5:
**MspJI (a), FspEI (b) and LpnPI (c)-enzymatic digestion of 5** ^**m**^ **C-containing long PCR amplicons.** Molar concentration denotes the 5-methyl-dCTP-concentration in PCR solution. Additional file 6:
**Information on** ***Arabidopsis thaliana*** **genome-derived BAC clones used for short-read sequencing.**
Additional file 7:
**Cost assumption (US$) for Sanger method-based BAC-end sequencing, and Φ29 and MspJI-based short-read sequencing.** Cost for BAC-end sequencing was calculated based on previous publications [13]. Additional file 8:
**Illumina MiSeq sequencing library constructed with the MspJI-digested PCR amplicons.** A peak between the lower and upper markers represents the size distribution of DNA fragments of the constructed DNA library. Additional file 9:
**Genetic linkage mapping result of perennial ryegrass flowering signaling pathway-related sequences.** The linkage maps were generated with the JoinMap® 3.0 program. The *Lp*FLD, *Lp*Vrn5, *Lp*TOC1, and *Lp*LHY loci were assigned to linkage groups (LGs) 2, 5, 6, and 7, respectively. Two loci (*Lp*AP1 and *Lp*PHY) were assigned to LG4. The loci mapped through the MspJI-based fragmentation method are indicated in red. Additional file 10:
**Cost assumption for Illumina sequencing library preparation.** Cost (US$) for major kit, reagent, consumable items and capital equipment was calculated based on previous publications [22,23]. Additional file 11:
**Time period requirement assumption for Illumina sequencing library preparation.** Time periods required for library construction with the Covaris equipment-based and MspJI-based DNA fragmentation methods were estimated based on previous publications [22].
