# Supplementary material for: A simple method for semi-random DNA amplicon fragmentation using the methylation-dependent restriction enzyme MspJI
Source: BMC Biotechnol. 2015 Apr 11;15:25. doi: 10.1186/s12896-015-0139-7 (PMC4396059; doi:10.1186/s12896-015-0139-7)
Supplement: Additional file 2: — MspJI-enzymatic digestion of 5 m C-containing PCR amplicons. The Agro_gc50 sequence was amplified with the Immolase™, MangoTaq™ Pfu and Phusion Hot Start polymerases and digested with MspJI. Molar concentration denotes the 5-methyl-dCTP-concentration in the PCR solution. [file 12896_2015_139_MOESM2_ESM.pptx]

## Slide 1
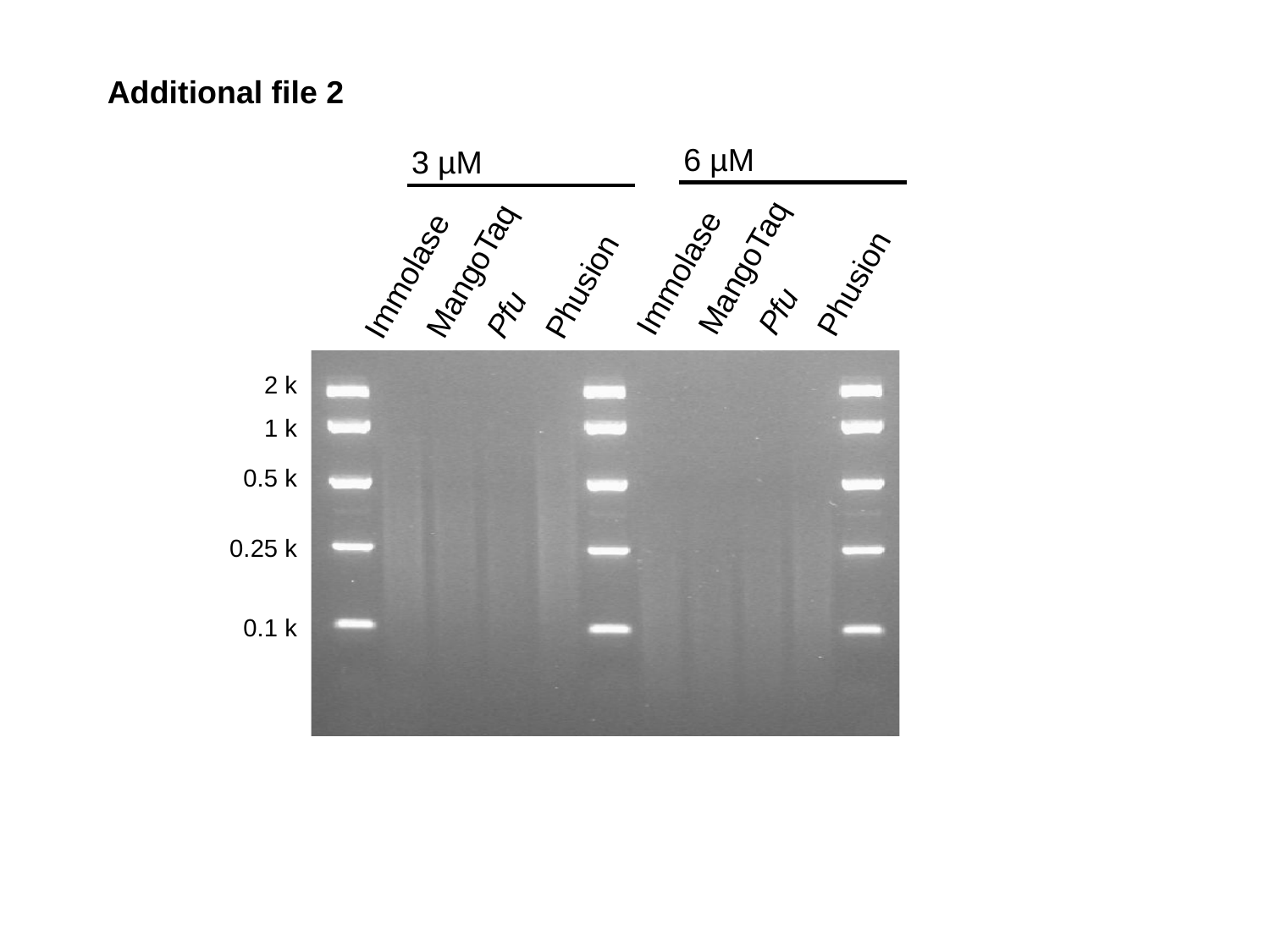

Additional file 2
6 µM
3 µM
MangoTaq
Pfu
Immolase
Phusion
MangoTaq
Pfu
Immolase
Phusion
2 k
1 k
0.5 k
0.25 k
0.1 k
