# Supplementary figures and images for: A simple method for semi-random DNA amplicon fragmentation using the methylation-dependent restriction enzyme MspJI
Source: BMC Biotechnol. 2015 Apr 11;15:25. doi: 10.1186/s12896-015-0139-7 (PMC4396059; doi:10.1186/s12896-015-0139-7)

## Slide 1
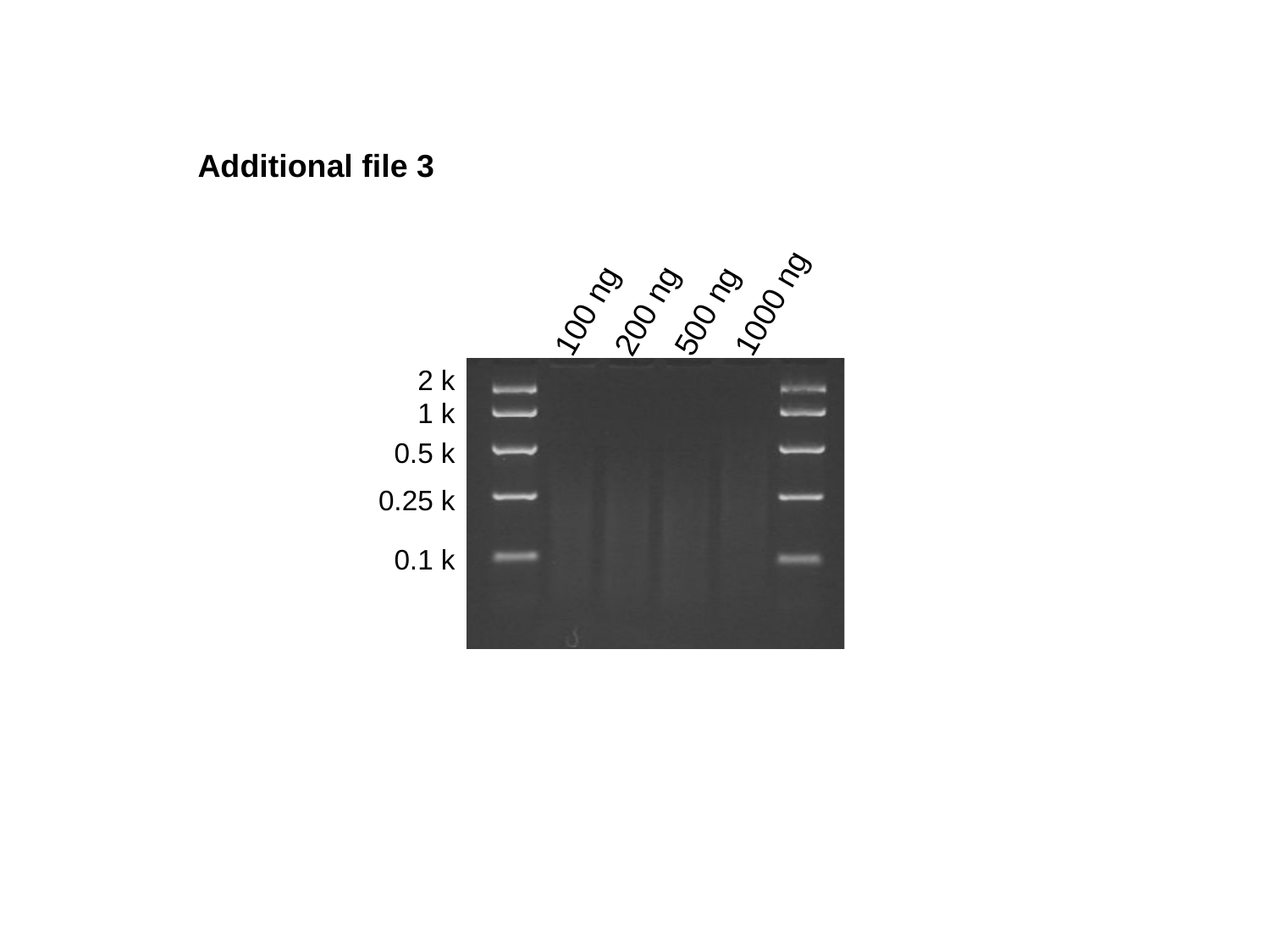

Additional file 3
100 ng
200 ng
500 ng
1000 ng
2 k
1 k
0.5 k
0.25 k
0.1 k

Supplement: Additional file 3: — Characterisation of MspJI enzymatic activity. Through PCR, 8 μM 5mC-containing amplicons were generated, and the products were cleaned with the AMPure kit. A range of DNA amounts (100, 200, 500, and 1,000 ng) was used for MspJI digestion, of which 50 ng digested DNA was visualised on an agarose gel. [file 12896_2015_139_MOESM3_ESM.pptx]

## Slide 1
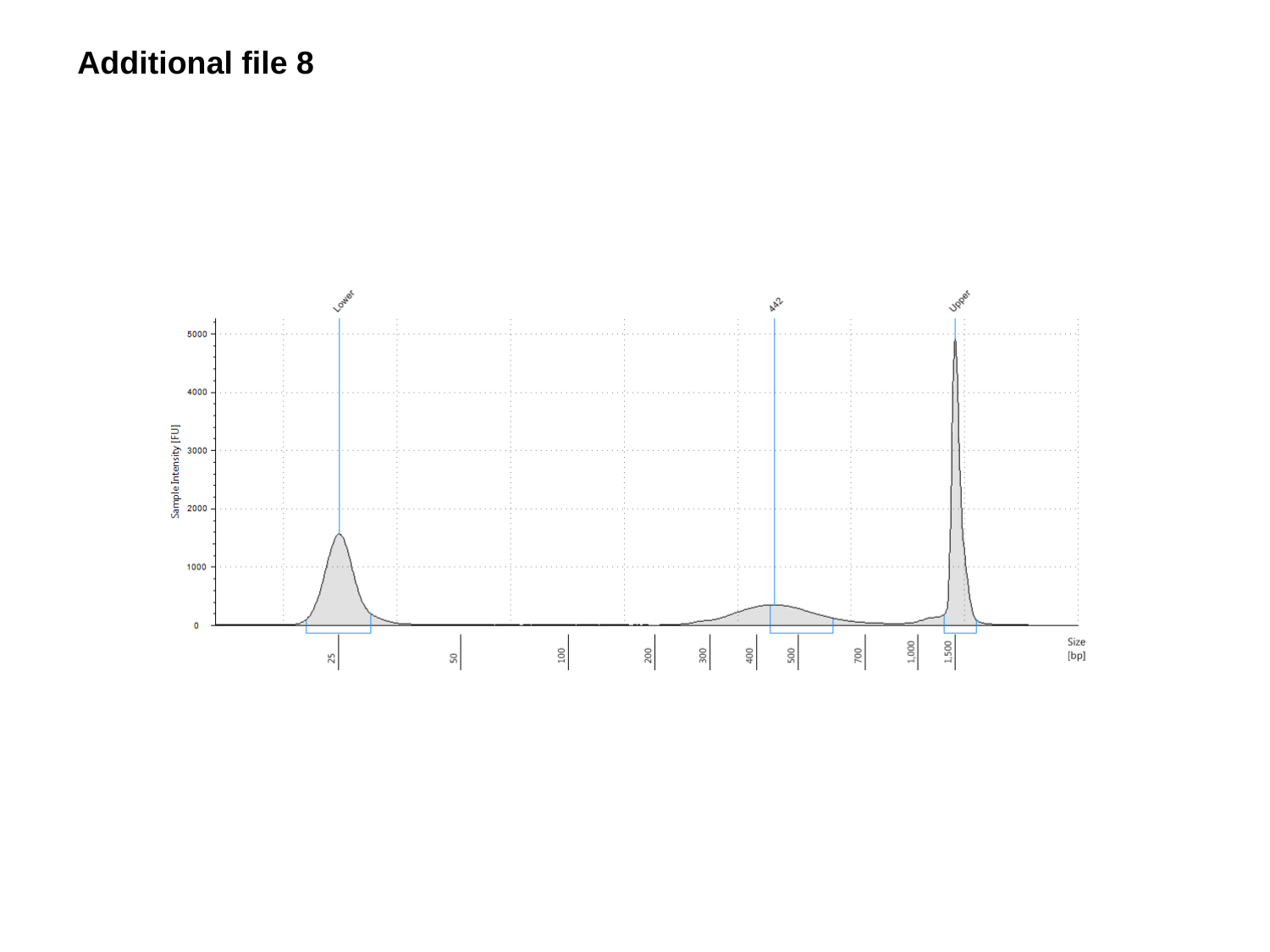

Additional file 8

Supplement: Additional file 8: — Illumina MiSeq sequencing library constructed with the MspJI-digested PCR amplicons. A peak between the lower and upper markers represents the size distribution of DNA fragments of the constructed DNA library. [file 12896_2015_139_MOESM8_ESM.pptx]
