# Supplementary material for: A simple method for semi-random DNA amplicon fragmentation using the methylation-dependent restriction enzyme MspJI
Source: BMC Biotechnol. 2015 Apr 11;15:25. doi: 10.1186/s12896-015-0139-7 (PMC4396059; doi:10.1186/s12896-015-0139-7)
Supplement: Additional file 4: — Illumina MiSeq sequencing libraries constructed with the MspJI, FspEI or LpnPI-digested amplicons. Sharp peaks with red and blue triangles show size standards (15 bp and 1,500 bp, respectively) of the Agilent DNA 1000 Kit. A peak between 200 and 1,500 bp represents the size distribution of DNA fragments of the constructed DNA library. [file 12896_2015_139_MOESM4_ESM.pptx]

## Slide 1
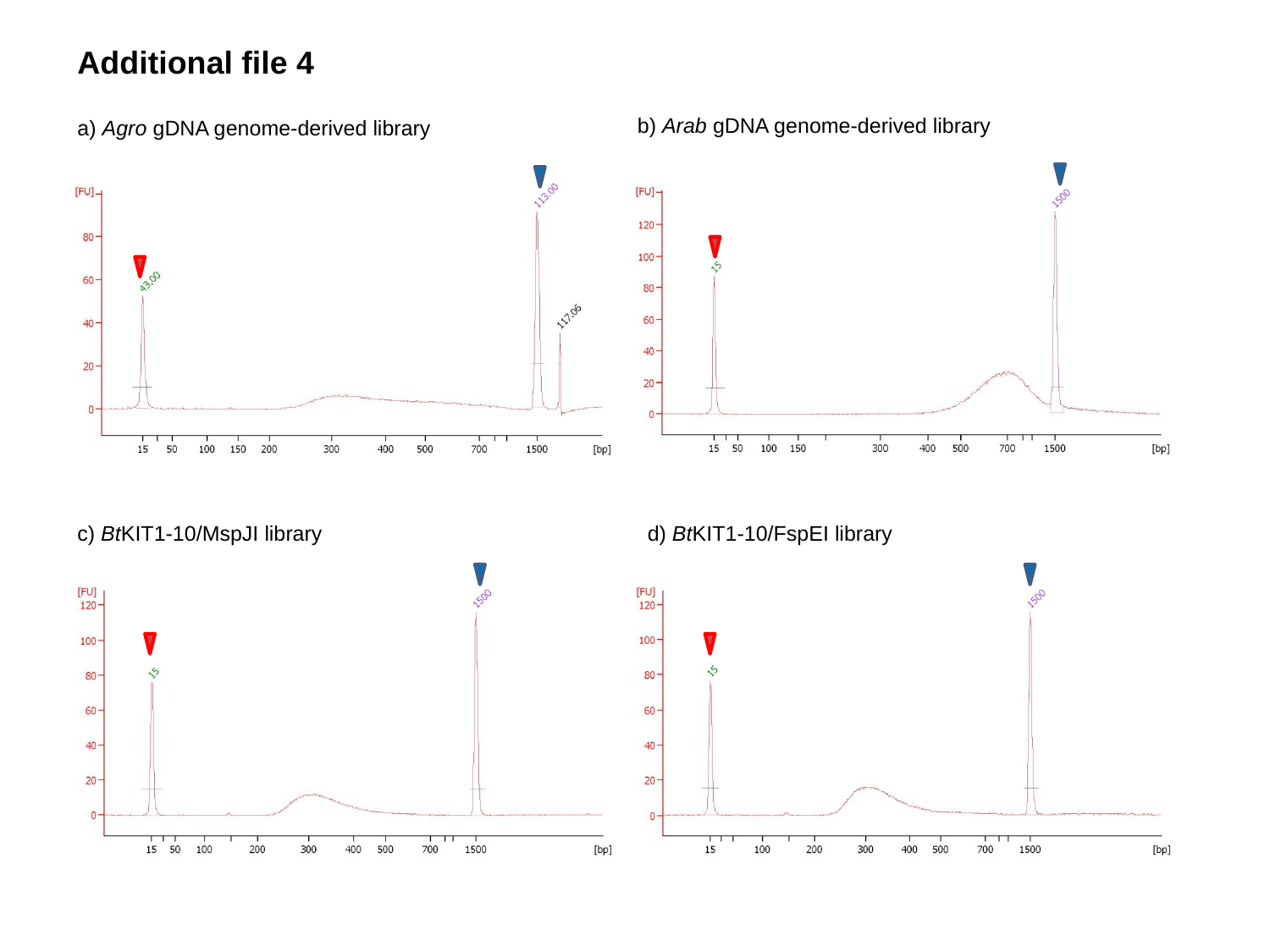

Additional file 4
b) Arab gDNA genome-derived library
a) Agro gDNA genome-derived library
c) BtKIT1-10/MspJI library
d) BtKIT1-10/FspEI library

## Slide 2
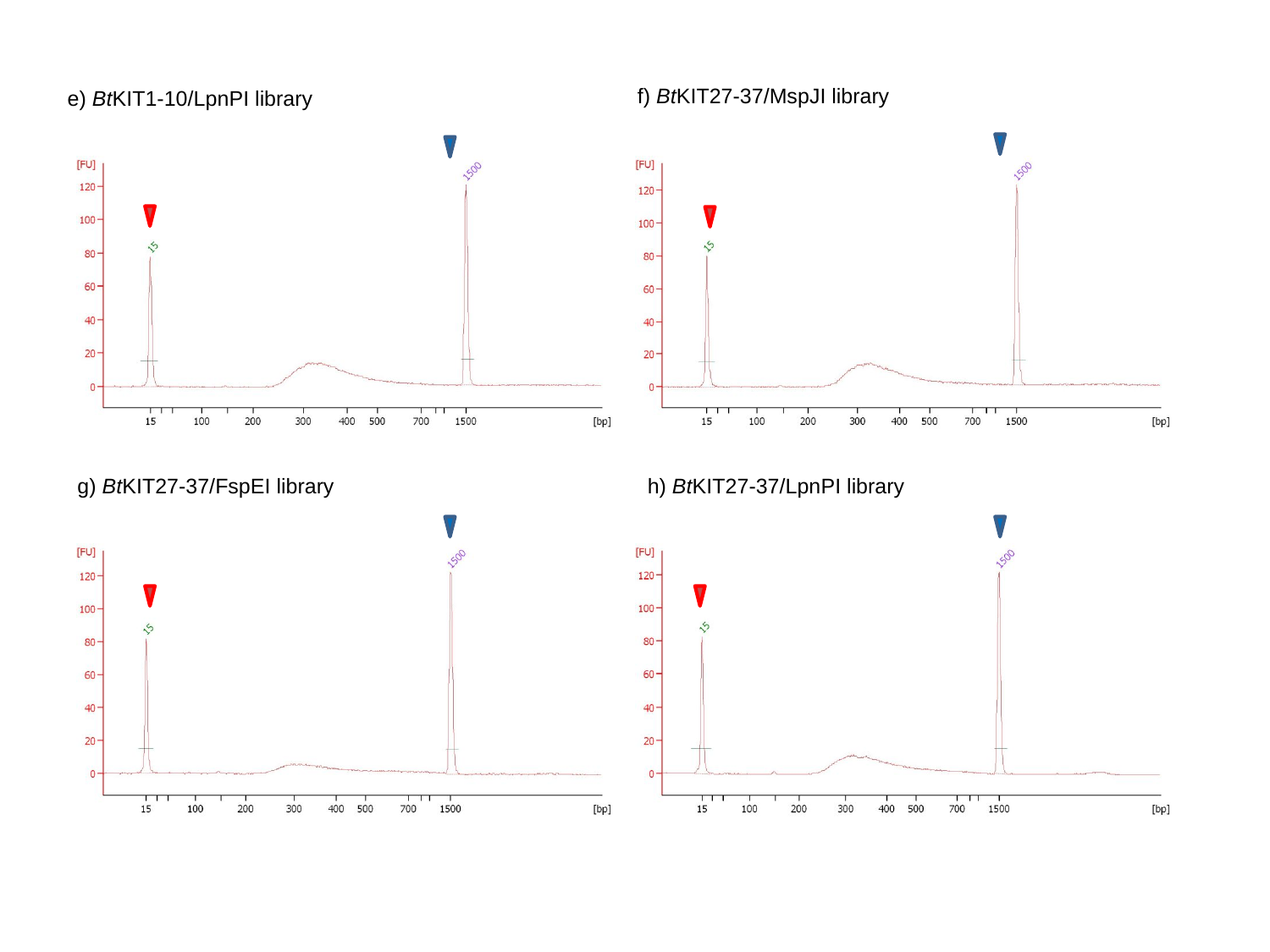

f) BtKIT27-37/MspJI library
e) BtKIT1-10/LpnPI library
g) BtKIT27-37/FspEI library
h) BtKIT27-37/LpnPI library

## Slide 3
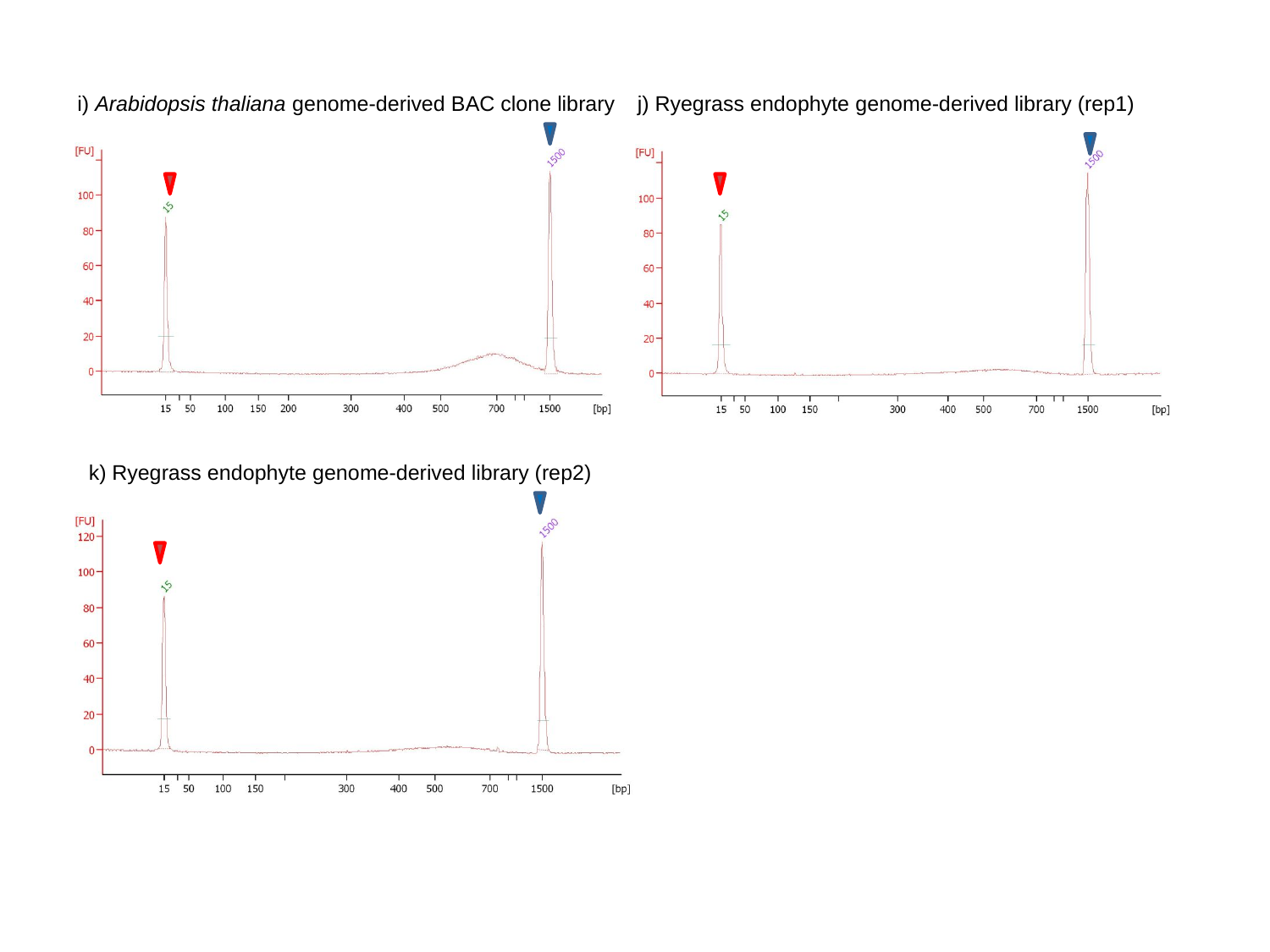

i) Arabidopsis thaliana genome-derived BAC clone library
j) Ryegrass endophyte genome-derived library (rep1)
k) Ryegrass endophyte genome-derived library (rep2)
