# Supplementary material for: A simple method for semi-random DNA amplicon fragmentation using the methylation-dependent restriction enzyme MspJI
Source: BMC Biotechnol. 2015 Apr 11;15:25. doi: 10.1186/s12896-015-0139-7 (PMC4396059; doi:10.1186/s12896-015-0139-7)
Supplement: Additional file 5: — MspJI (a), FspEI (b) and LpnPI (c)-enzymatic digestion of 5 m C-containing long PCR amplicons. Molar concentration denotes the 5-methyl-dCTP-concentration in PCR solution. [file 12896_2015_139_MOESM5_ESM.pptx]

## Slide 1
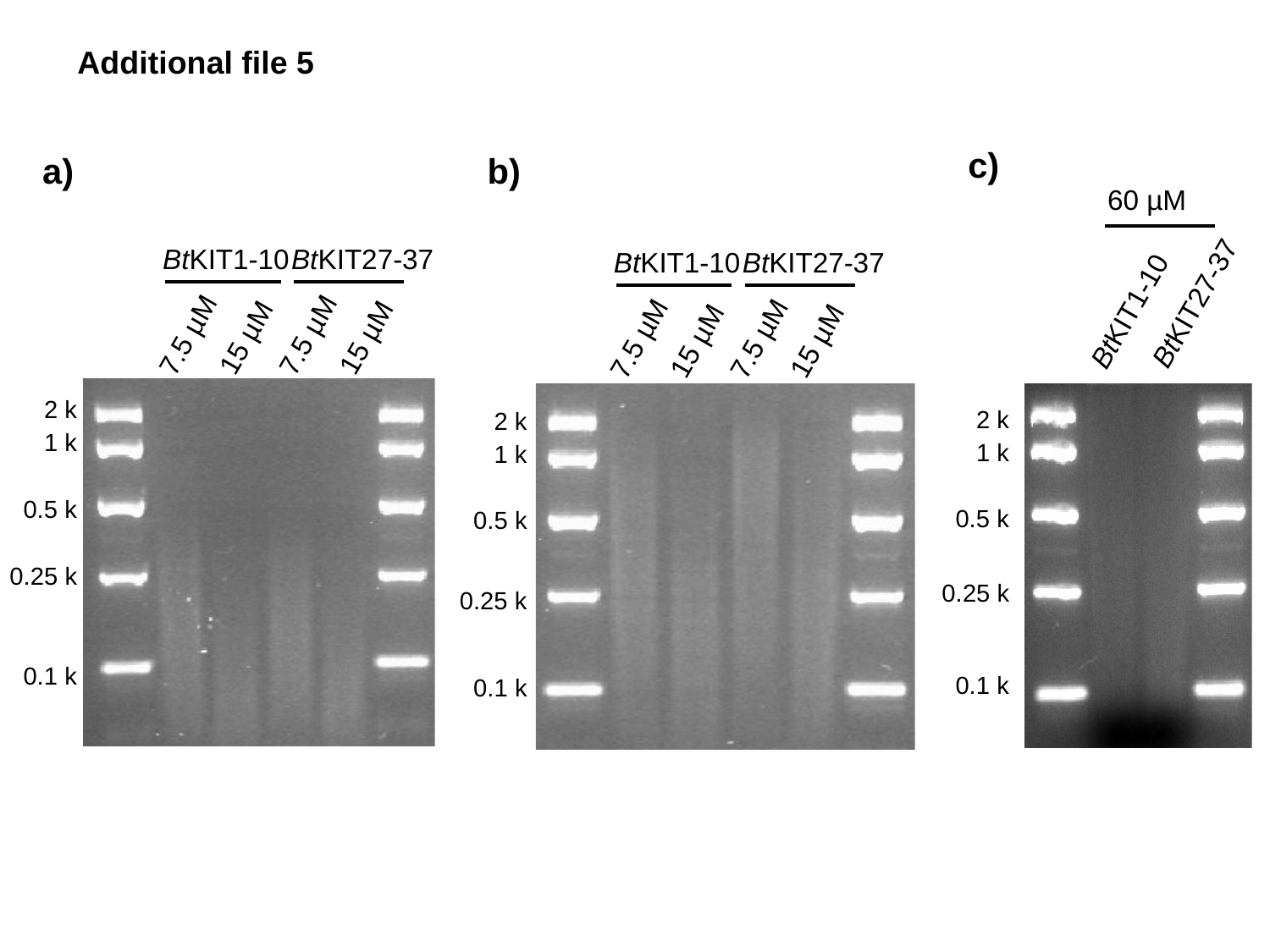

Additional file 5
c)
a)
b)
60 µM
BtKIT1-10
BtKIT27-37
BtKIT1-10
BtKIT27-37
BtKIT27-37
BtKIT1-10
7.5 µM
7.5 µM
15 µM
15 µM
7.5 µM
7.5 µM
15 µM
15 µM
2 k
2 k
2 k
1 k
1 k
1 k
0.5 k
0.5 k
0.5 k
0.25 k
0.25 k
0.25 k
0.1 k
0.1 k
0.1 k
