# Supplementary material for: A simple method for semi-random DNA amplicon fragmentation using the methylation-dependent restriction enzyme MspJI
Source: BMC Biotechnol. 2015 Apr 11;15:25. doi: 10.1186/s12896-015-0139-7 (PMC4396059; doi:10.1186/s12896-015-0139-7)
Supplement: Additional file 9: — Genetic linkage mapping result of perennial ryegrass flowering signaling pathway-related sequences. The linkage maps were generated with the JoinMap® 3.0 program. The LpFLD, LpVrn5, LpTOC1, and LpLHY loci were assigned to linkage groups (LGs) 2, 5, 6, and 7, respectively. Two loci (LpAP1 and LpPHY) were assigned to LG4. The loci mapped through the MspJI-based fragmentation method are indicated in red. [file 12896_2015_139_MOESM9_ESM.pptx]

## Slide 1
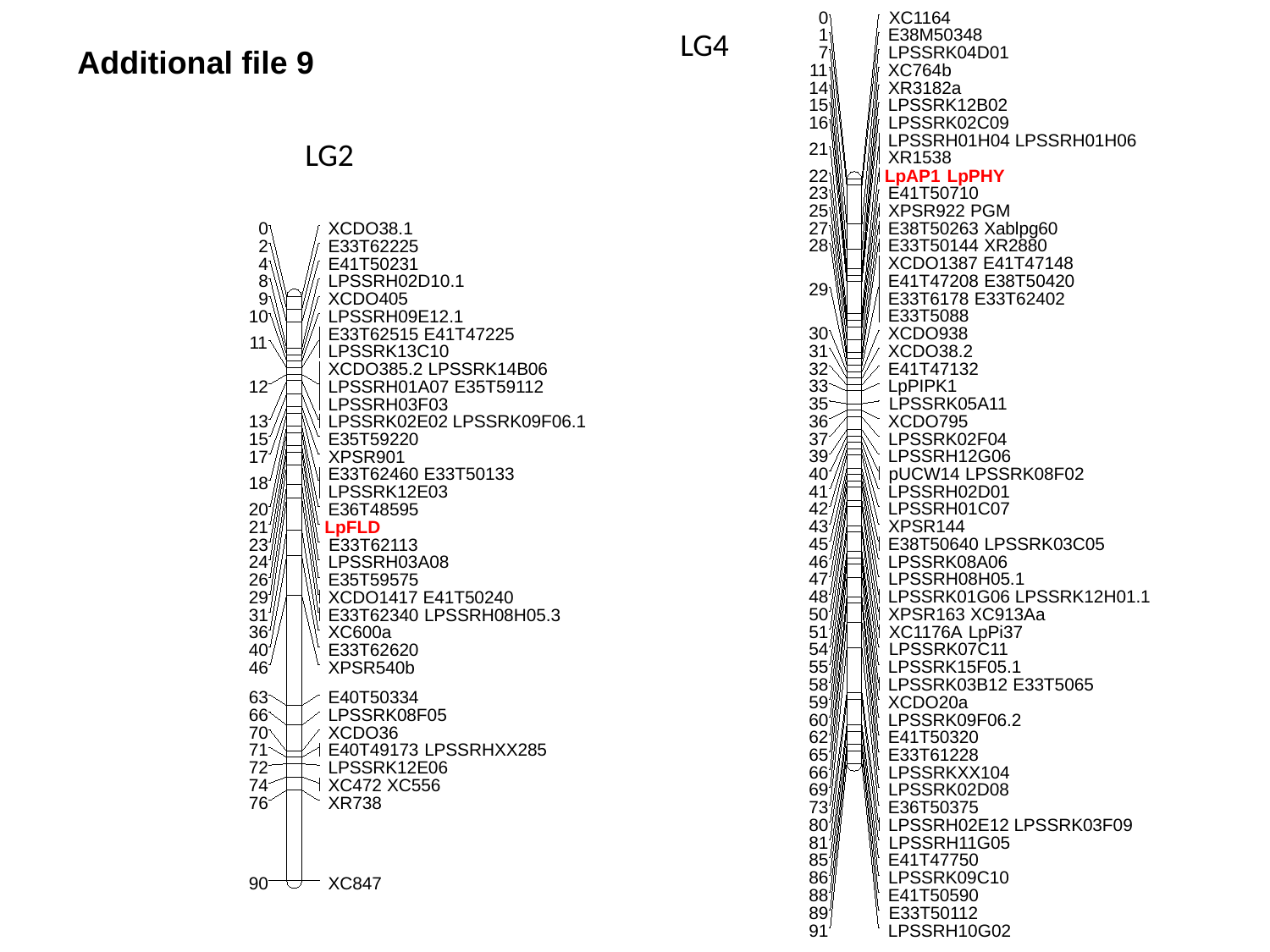

0
XC1164
LG4
1
E38M50348
Additional file 9
7
LPSSRK04D01
11
XC764b
14
XR3182a
15
LPSSRK12B02
16
LPSSRK02C09
LG2
LPSSRH01H04
LPSSRH01H06
21
XR1538
22
LpAP1
LpPHY
23
E41T50710
25
XPSR922
PGM
27
E38T50263
Xablpg60
0
XCDO38.1
28
E33T50144
XR2880
2
E33T62225
XCDO1387
E41T47148
4
E41T50231
E41T47208
E38T50420
8
LPSSRH02D10.1
29
E33T6178
E33T62402
9
XCDO405
E33T5088
10
LPSSRH09E12.1
30
XCDO938
E33T62515
E41T47225
11
31
XCDO38.2
LPSSRK13C10
32
E41T47132
XCDO385.2
LPSSRK14B06
33
LpPIPK1
12
LPSSRH01A07
E35T59112
35
LPSSRK05A11
LPSSRH03F03
36
XCDO795
13
LPSSRK02E02
LPSSRK09F06.1
37
LPSSRK02F04
15
E35T59220
39
LPSSRH12G06
17
XPSR901
40
pUCW14
LPSSRK08F02
E33T62460
E33T50133
18
41
LPSSRH02D01
LPSSRK12E03
42
LPSSRH01C07
20
E36T48595
43
XPSR144
21
LpFLD
45
E38T50640
LPSSRK03C05
23
E33T62113
46
LPSSRK08A06
24
LPSSRH03A08
47
LPSSRH08H05.1
26
E35T59575
48
LPSSRK01G06
LPSSRK12H01.1
29
XCDO1417
E41T50240
50
XPSR163
XC913Aa
31
E33T62340
LPSSRH08H05.3
51
XC1176A
LpPi37
36
XC600a
54
LPSSRK07C11
40
E33T62620
55
LPSSRK15F05.1
46
XPSR540b
58
LPSSRK03B12
E33T5065
63
E40T50334
59
XCDO20a
66
LPSSRK08F05
60
LPSSRK09F06.2
70
XCDO36
62
E41T50320
71
E40T49173
LPSSRHXX285
65
E33T61228
72
LPSSRK12E06
66
LPSSRKXX104
74
XC472
XC556
69
LPSSRK02D08
76
XR738
73
E36T50375
80
LPSSRH02E12
LPSSRK03F09
81
LPSSRH11G05
85
E41T47750
86
LPSSRK09C10
90
XC847
88
E41T50590
89
E33T50112
91
LPSSRH10G02

## Slide 2
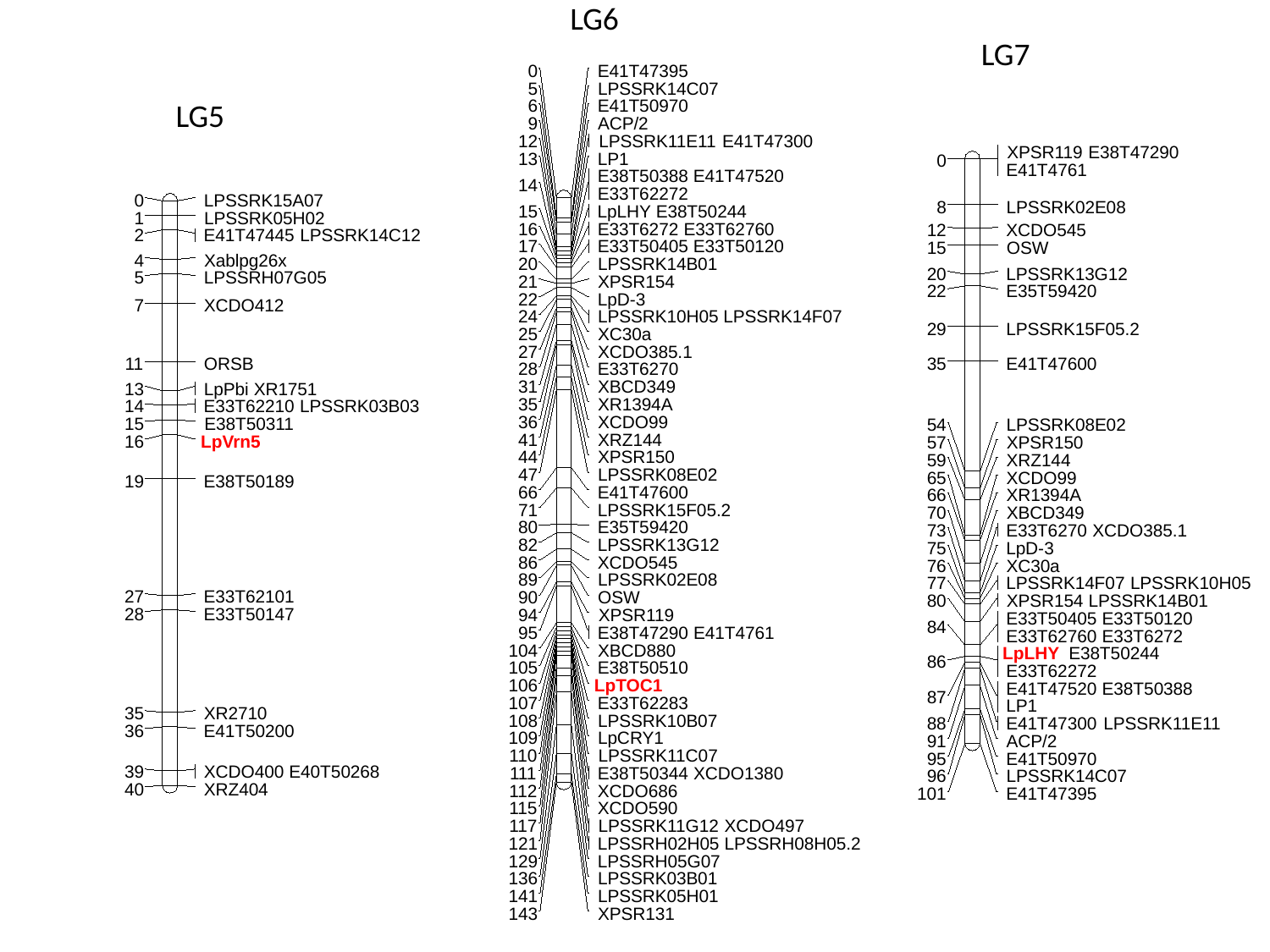

LG6
LG7
0
E41T47395
5
LPSSRK14C07
LG5
6
E41T50970
9
ACP/2
12
LPSSRK11E11
E41T47300
XPSR119
E38T47290
13
LP1
0
E41T4761
E38T50388
E41T47520
14
E33T62272
0
LPSSRK15A07
8
LPSSRK02E08
15
LpLHY
E38T50244
1
LPSSRK05H02
16
E33T6272
E33T62760
12
XCDO545
2
E41T47445
LPSSRK14C12
17
E33T50405
E33T50120
15
OSW
4
Xablpg26x
20
LPSSRK14B01
20
LPSSRK13G12
5
LPSSRH07G05
21
XPSR154
22
E35T59420
22
LpD-3
7
XCDO412
24
LPSSRK10H05
LPSSRK14F07
29
LPSSRK15F05.2
25
XC30a
27
XCDO385.1
35
E41T47600
11
ORSB
28
E33T6270
31
XBCD349
13
LpPbi
XR1751
35
XR1394A
14
E33T62210
LPSSRK03B03
36
XCDO99
15
E38T50311
54
LPSSRK08E02
41
XRZ144
16
LpVrn5
57
XPSR150
44
XPSR150
59
XRZ144
47
LPSSRK08E02
65
XCDO99
19
E38T50189
66
E41T47600
66
XR1394A
71
LPSSRK15F05.2
70
XBCD349
80
E35T59420
73
E33T6270
XCDO385.1
82
LPSSRK13G12
75
LpD-3
86
XCDO545
76
XC30a
89
LPSSRK02E08
77
LPSSRK14F07
LPSSRK10H05
27
E33T62101
90
OSW
80
XPSR154
LPSSRK14B01
28
E33T50147
94
XPSR119
E33T50405
E33T50120
84
95
E38T47290
E41T4761
E33T62760
E33T6272
104
XBCD880
LpLHY
E38T50244
86
105
E38T50510
E33T62272
106
LpTOC1
E41T47520
E38T50388
87
107
E33T62283
LP1
35
XR2710
108
LPSSRK10B07
88
E41T47300
LPSSRK11E11
36
E41T50200
109
LpCRY1
91
ACP/2
110
LPSSRK11C07
95
E41T50970
39
XCDO400
E40T50268
111
E38T50344
XCDO1380
96
LPSSRK14C07
40
XRZ404
112
XCDO686
101
E41T47395
115
XCDO590
117
LPSSRK11G12
XCDO497
121
LPSSRH02H05
LPSSRH08H05.2
129
LPSSRH05G07
136
LPSSRK03B01
141
LPSSRK05H01
143
XPSR131
